# Upregulation of Transglutaminase and *ε* (*γ*-Glutamyl)-Lysine in the Fisher-Lewis Rat Model of Chronic Allograft Nephropathy

**DOI:** 10.1155/2014/651608

**Published:** 2014-07-21

**Authors:** Badri Shrestha, Imran Butt, Michelle Da Silva, Armando Sanchez-Lara, Bart Wagner, Andrew Raftery, Timothy Johnson, John Haylor

**Affiliations:** ^1^Division of Renal Transplantation, Sheffield Kidney Institute, Northern General Hospital, Herries Road, Sheffield S5 7AU, UK; ^2^Academic Nephrology Unit, Medical School, University of Sheffield, Sheffield S10 2RX, UK

## Abstract

*Background*. Tissue transglutaminase (TG2), a cross-linking enzyme, modulates deposition of extracellular matrix protein in renal fibrosis. This study aimed to examine TG2 and its cross-link product *ε*(*γ*-glutamyl)-lysine in the Fisher-Lewis rat renal transplantation (RTx) model of chronic allograft nephropathy (CAN). *Materials and Methods*. Left renal grafts from male Fisher and Lewis were transplanted into Lewis rats, generating allografts and isografts, respectively. Blood pressure, renal function, and proteinuria were monitored for up to 52 weeks. At termination, CAN was assessed in the renal tissue by light and electron microscopy, TG2 and *ε*(*γ*-glutamyl)-lysine by immunofluorescence, and the urinary *ε*(*γ*-glutamyl)-lysine by high performance liquid chromatography. *Results*. Compared to the isograft, the allografts were hypertensive, proteinuric, and uraemic and developed CAN. Extracellular TG2 (glomerulus: 64.55 ± 17.61 versus 2.11 ± 0.17, *P* < 0.001; interstitium: 13.72 ± 1.62 versus 3.19 ± 0.44, *P* < 0.001), *ε*(*γ*-glutamyl)-lysine (glomerulus: 21.74 ± 2.71 versus 1.98 ± 0.37, *P* < 0.01; interstitium: 37.96 ± 17.06 versus 0.42 ± 0.11, *P* < 0.05), TG2 enzyme activity (1.09 ± 0.13 versus 0.41 ± 0.03 nmol/h/mg protein, *P* < 0.05), TG2 mRNA (20-fold rise), and urinary *ε*(*γ*-glutamyl)-lysine (534.2 ± 198.4 nmol/24 h versus 57.2 ± 4.1 nmol/24 h, *P* < 0.05) levels were significantly elevated in the allografts and showed a positive linear correlation with tubulointerstitial fibrosis. *Conclusion*. CAN was associated with upregulation of renal TG2 pathway, which has a potential for pharmacological intervention. The elevated urinary *ε*(*γ*-glutamyl)-lysine, measured for the first time in RTx, is a potential biomarker of CAN.

## 1. Introduction

The advantages conferred by renal transplantation (RTx), such as the improved quality of life [1] and survival, are continually compromised by graft loss due to development of chronic allograft nephropathy (CAN), which manifests with hypertension, proteinuria, and progressive deterioration of renal function due to interstitial fibrosis (IF), tubular atrophy (TA), obliterative arteriolopathy, and glomerulosclerosis [2], which is initiated by the interplay of alloantigen-dependent and alloantigen-independent risk factors [3, 4]. Hypotheses propounded to explain its pathogenesis include chronic antibody-mediated rejection, input-stress model, replicative senescence, cytokine excess, cumulative damage, and oxidative stress [5–11] leading to epithelial-mesenchymal transdifferentiation (EMT) and the elaboration of the extracellular matrix (ECM) in the tubulointerstitial, glomerular, and vascular compartments of the kidney [12, 13].

In the early stage of CAN, renal function can be preserved by control of hypertension [14], proteinuria [15], hyperlipidaemia [16], and institution of least nephrotoxic immunosuppressive regimens [17]. However, as yet, established CAN has no treatment due, in part, to irreversible tissue damage at the time of diagnosis. ECM deposition is a key event in the development of CAN and targeting graft fibrosis has recently been proposed as a new focus in organ transplantation [13].

**Figure 1:**
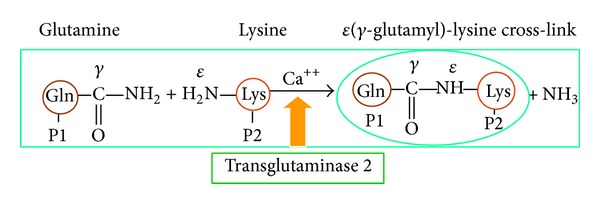
Glutamine (P1) and lysine (P2) residues of proteins are linked together by TG2 in presence of Ca^+2^ leading to the formation of *ε*(*γ*-glutamyl)-lysine cross-link.

Tissue transglutaminase (TG2) (EC 2.3.2.13) catalyses Ca^+2^-dependent, transamidation reactions by the transfer of acyl groupings from glutamine to lysine residues of ECM protein molecules forming a covalent bond, where the cross-link product, *ε*(*γ*-glutamyl)-lysine, leads to ECM stabilisation (Figure 1) [18, 19]. TG2 is activated, following its export from the cell, by calcium in the extracellular fluid. Activated TG2 accelerates the deposition of ECM components, its cross-linked products being highly resistant to proteolytic degradation by matrix metalloproteinases (MMPs) [20]. Excessive expansion of ECM leads to cellular damage mainly from ischaemia. TG2 is also involved in cellular apoptosis [21]. Significant upregulation of TG2 has previously been observed in experimental rat models of 5/6 subtotal nephrectomy [21] and diabetic nephropathy [22] as well as human renal disease [23] and human RTx biopsies of CAN [24]. Literature search showed absence of studies of TG2 in animal experimental models of RTx, hence making it a new tool for interventional studies in the prevention of CAN. Likewise, there is no record of estimation of cross-link *ε*(*γ*-glutamyl)-lysine in the urine samples in human or animal models of transplantation in relation to transplant fibrosis.

The aim of the present study was to examine the association of TG2 pathway with the CAN in an allogenic Fisher-Lewis rat RTx model and to measure urinary cross-link to assess its relationship with development of CAN.

## 2. Materials and Methods

### 2.1. Animals

Male inbred Fisher (F334, RT1^1v1^) and Lewis (LEW, RT1^1^) rats weighing 250–300 g were purchased from Harlan, UK. All experiments were performed in accordance with the protocols of the Animals (Scientific Procedures) Act 1986 and approval from the Home Office (UK).

### 2.2. Rat Renal Transplant Model

The left donor kidney was retrieved from the Fisher or Lewis donor rats and transplanted into Lewis rats, thereby generating the Fisher-Lewis allografts and Lewis-Lewis isografts, respectively. Under isoflurane inhalation anaesthesia, through a midline incision, the left kidney was retrieved with a segment of aorta and inferior venacava and their upper ends were ligated with 3/0 vicryl. The kidneys were perfused in situ with University of Wisconsin solution by cannulation of infrarenal aorta and preserved in the same solution at 4°C. In the recipients, left native nephrectomy and skeletonisation of abdominal aorta and inferior venacava were performed. With the aid of an operating microscope, an end-to-side anastomosis was performed between the aortic and inferior venacaval (IVC) conduits of the donor kidney to the recipient abdominal aorta and IVC, respectively, using 10/0 prolene suture. The bladder cuff attached to the donor ureter was anastomosed to the dome of the recipient bladder. Intravenous fluids were administered during both donor and recipient surgery to maintain systemic blood pressure ensuring adequate perfusion of the donor kidney and adequate reperfusion of the recipient kidney. All recipient rats were given cyclosporine (Novartis, UK) 5 mg/kg s.c. for 10 days, which was followed by right native nephrectomy.

Transplanted kidneys were harvested when an animal's physical condition indicated the development of uraemia, as reflected by a decrease in body weight and physical condition in accordance with Home Office Project License or after 52 weeks. The average study duration for the isografts was 51 weeks (*n* = 5) and for the allografts 42 weeks (*n* = 7) without reaching a statistical difference. Portions of the kidneys were snap frozen in liquid nitrogen and stored at −150°C for immunohistochemical staining and messenger ribonucleic acid (mRNA). Other samples were fixed in 4% formalin and 3% phosphate buffered glutaraldehyde for histological and electron microscopic evaluation, respectively.

### 2.3. Functional Measurements

Serum creatinine, creatinine clearance, protein excretion, and blood pressure were measured at 8 weekly intervals as described previously [25]. Albuminuria was measured by a rat albumin ELISA (enzyme-linked immunosorbent analysis) (BiogNosis, Halisham, UK).

### 2.4. Histology

Formalin-fixed, paraffin-embedded tissues were sectioned, stained with Masson's trichrome, examined by light microscopy, and analysed by multiphase image analysis [26]. The tubular scarring index was determined as a ratio of extracellular matrix to cell volume. Tubular dilation was assessed from the tubular lumen area and tubular atrophy was assessed from the tubular cell area. Kidney tissue stored in 3% phosphate buffered glutaraldehyde was fixed in 1% aqueous osmium tetroxide, blocked in epoxy resin, and subjected to transmission electron microscopy [27].

### 2.5. TG2 Activity Assay

Total TG2 enzyme activity was measured by calcium (Ca^2+^) dependent incorporation of [1,4,-^14^C] putrescine into N′,N′′-dimethylcasein. The tissue homogenates were incubated (20 minutes, 37°C) with [1,4,-^14^C] putrescine (2.5 nM, 3.97 mice/mmol; Amersham International, Little Chalfont, UK), dithiothreitol (3.8 mM), calcium chloride (2.5 mM), and dimethylcasein (5 mg/mL). Homogenate was spotted onto 1 cm^2^ filter papers and precipitated with ice-cold 10% (wt/vol) trichloroacetic acid. After washing, the putrescine incorporation into precipitated protein was determined by scintillation counting. Results were then corrected to U/mg/protein. 1 unit (U) of activity was equivalent to 1 nmol putrescine incorporated per hour at 37°C [28].

### 2.6. Immunolocalisation of TG2 and *ε*(*γ*-Glutamine)-Lysine Cross-Link

The distribution of immunoreactive TG2 and *ε*(*γ*-glutamine)-lysine cross-link was determined by immunohistochemistry and quantified by immunofluorescence. 10 *μ*m cryostat sections obtained from tissue stored in liquid nitrogen were cut (−12°C), air dried (37°C), and blocked in washing buffer (10 mM EDTA, protease inhibitor cocktail, 5% BSA, 0.01% triton X-100 + 5% normal horse serum) for 60 minutes at room temperature. Washed sections were incubated with a primary monoclonal, mouse antibody at a dilution of 1 : 20 for TG2 (TgII Ab-2, TG100, Neomakers, Fremont, CA, USA) for 12 hours at 4°C. The sections were fixed in ice cold acetone for 5 minutes and incubated for 2 hours with an anti-mouse secondary antibody (1/50 dilution in PBS containing 3% BSA) tagged with fluorescein IgG (FITC) (T0116 (H+L). The sections were mounted in Vectashield hardest fluorescent mounting media with DAPI (H-1500, Vector, Burlingame, US).

For immunolocalisation of cross-link, a primary monoclonal mouse antibody at a dilution of 1 : 20 for *ε*(*γ*-glutamyl)-lysine (clone 81D4, Covalab, Lyon, France) and an anti-mouse secondary antibody (1/50 dilution in PBS containing 3% BSA) tagged with fluorescein IgG (FITC) (T0116 (H+L), Vector, UK) were used by following the steps described above.

### 2.7. Quantification of TG2 and *ε*(*γ*-Glutamyl)-Lysine Cross-Link

Quantification was performed by viewing on a fluorescence Olympus BX61 microscope. Either 10 glomeruli (×400 magnification) or 10 overlapping tubulointerstitial fields were visualised (×100 magnification) per section, analysed by image analysis software (AnalySIS 3.2 software, Olympus Soft Imaging Systems, GmBH, Munster, Germany).

TG2 and cross-link fluorescence were quantified by three-phase image analysis. Results were expressed as a ratio of FITC stain (green) to nuclear DAPI stain (blue) areas.

### 2.8. Northern Blot

Total TG2 ribonucleic acid (RNA) was extracted using Trizol (Gibco, UK) and subjected to Northern blot analysis using the Bam 400 TG2 complementary deoxyribonucleic acid probe. Autoradiographs were quantified by scanning densitometry (Molecular Analyst version 4 software; Biorad, Hemel Hempstead, UK). Densitometry values were corrected for loading using the house keeping gene cyclophilin [29].

### 2.9. Urinary *ε*(*γ*-Glutamyl)-Lysine Cross Link

*ε*(*γ*-glutamyl)-lysine dipeptide was measured in urine following protein precipitation using 10% trichloroacetic acid in acetone and exhaustive proteolytic digestion by incubation with subtilisin, pronase E, carboxypeptidase (Sigma Aldrich, UK), and leucine amino peptidase and prolidase (NBS Biologicals, UK). Following fractionation of the freeze-dried residue after resuspension in lithium citrate and postcolumn derivatisation in ninhydrin, samples were read at 570 nm with a retention time of 77 minutes using cation-exchange high performance liquid chromatography (EzChomm Elite software; Biochrom, Cambridge, UK) using a reference to a 10 nm/20 *μ*L *ε*-(*γ*-glutamyl)-lysine calibration standard.

### 2.10. Statistical Analysis

Data analysis was carried out on an intention-to-treat basis and analysed and presented using GraphPad Prism 5 (GraphPad Software 2236, CA, USA). Values are presented as means ± S.E.M. Serial longitudinal measurements were compared by two-way ANOVA repeated measures (mixed model) with Bonferroni's post hoc test. Terminal tissue data was compared using the nonparametric Mann-Whitney *U* test. Significant difference was assigned when *P* < 0.05.

## 3. Results

### 3.1. Functional Measurements

**Figure 2:**
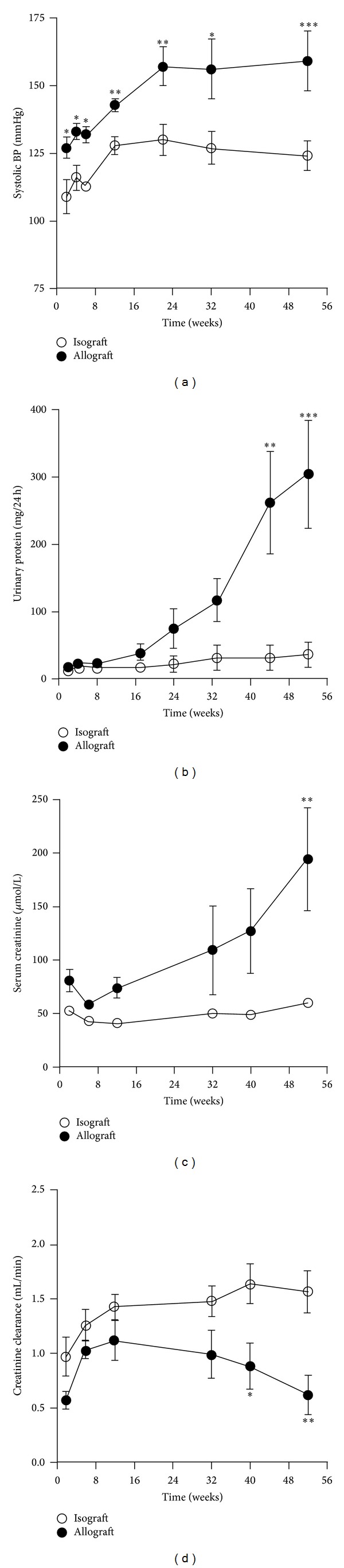
Serial measurements of (a) systolic blood pressure, (b) urinary protein, (c) serum creatinine, and (d) creatinine clearance for isografts and allografts. Results were expressed as mean ± SEM (*P < 0.05, **P < 0.01, and ***P < 0.001).

The allografts were hypertensive at 2 weeks post-RTx and had a higher systolic (Figure 2(a)), diastolic, and mean blood pressure than the isografts throughout the study. At termination, the systolic blood pressure was significantly higher in the allografts than the isografts (159 ± 11 versus 124 ± 5 mmHg, *P* < 0.01).

The isografts showed a slight increase in urinary protein excretion over the study period while the allografts showed a marked progressive rise in proteinuria of some 20-fold (Figure 2(b)). At termination, urinary protein excretion from the allografts was 8-fold higher than the isografts (303 ± 80 versus 37 ± 17 mg/24 h, *P* < 0.005) together with a 15-fold higher urinary excretion of albumin (60.7 ± 19 versus 4.1 ± 1.5 mg/24 h, *P* < 0.01).

Serum creatinine fell and creatinine clearance increased in the early post-RTx period in both isografts and allografts, reaching a maximum after 10 weeks (Figure 2(c)). In the isografts, creatinine clearance was stable for the remainder of the study. However, in the allografts creatinine clearance declined progressively (Figure 2(d)), together with a marked rise in serum creatinine. At termination, creatinine clearance in the allografts was approximately 30% of the creatinine clearance in the isografts (0.62 ± 0.18 versus 1.57 ± 0.19 mL/min, *P* < 0.01) together with a 3-fold higher serum creatinine (194 ± 48 versus 60 ± 5 *μ*mol/L, *P* < 0.01).

### 3.2. Masson's Trichrome Stain

**Figure 3:**
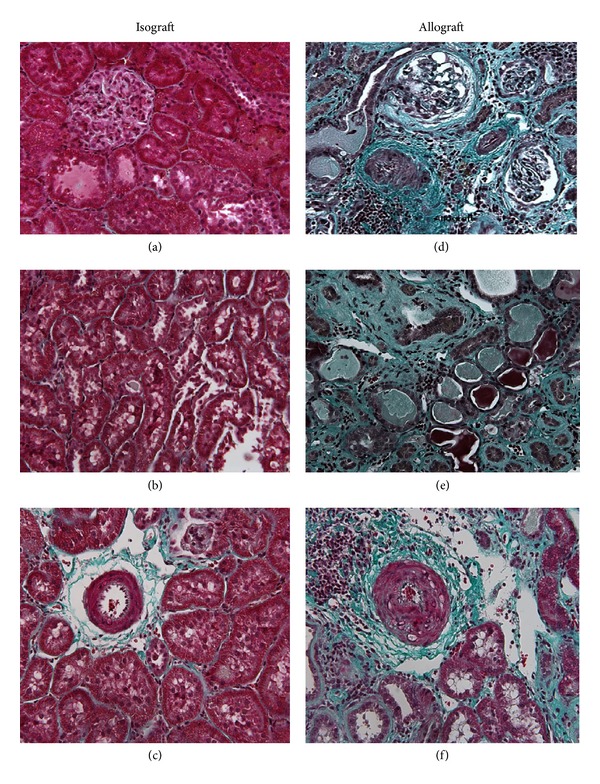
A representative field is shown from terminal kidney tissue of a glomerulus (a), the tubulointerstitium (b), and an arteriole (c) from an isograft and a glomerulus (d), the tubulointerstitium (e), and an arteriole (f) from an allograft. Sections were stained with Masson's trichrome and visualised using ×20 objective.

Representative fieldsof kidney sections stained with Masson's trichrome showed normal glomerular (Figure 3(a)) and tubular (Figure 3(b)) histology without any evidence of ECM expansion, tubular dilatation, or arteriolar intimal proliferation in isografts (Figure 3(c)). The allografts showed evidence of glomerulosclerosis (Figure 3(d)), a marked expansion of the ECM, and extensive tubular dilatation and atrophy (Figure 3(e)) together with concentric proliferation of the arteriolar intima (Figure 3(f)). The tubular scarring index was 15-fold higher in the allografts than the isografts (33.9 ± 7.5 versus 2.38 ± 0.18, *P* < 0.01). The allografts showed a 6-fold increase in the area of the tubular lumen (18.4 ± 4.2 versus 3.2 ± 0.4% field, *P* < 0.02) together with a marked decrease in the area of tubular cells (54 ± 5.1 versus 91.2 ± 0.7% field, *P* < 0.01).

### 3.3. Electron Microscopy

**Figure 4:**
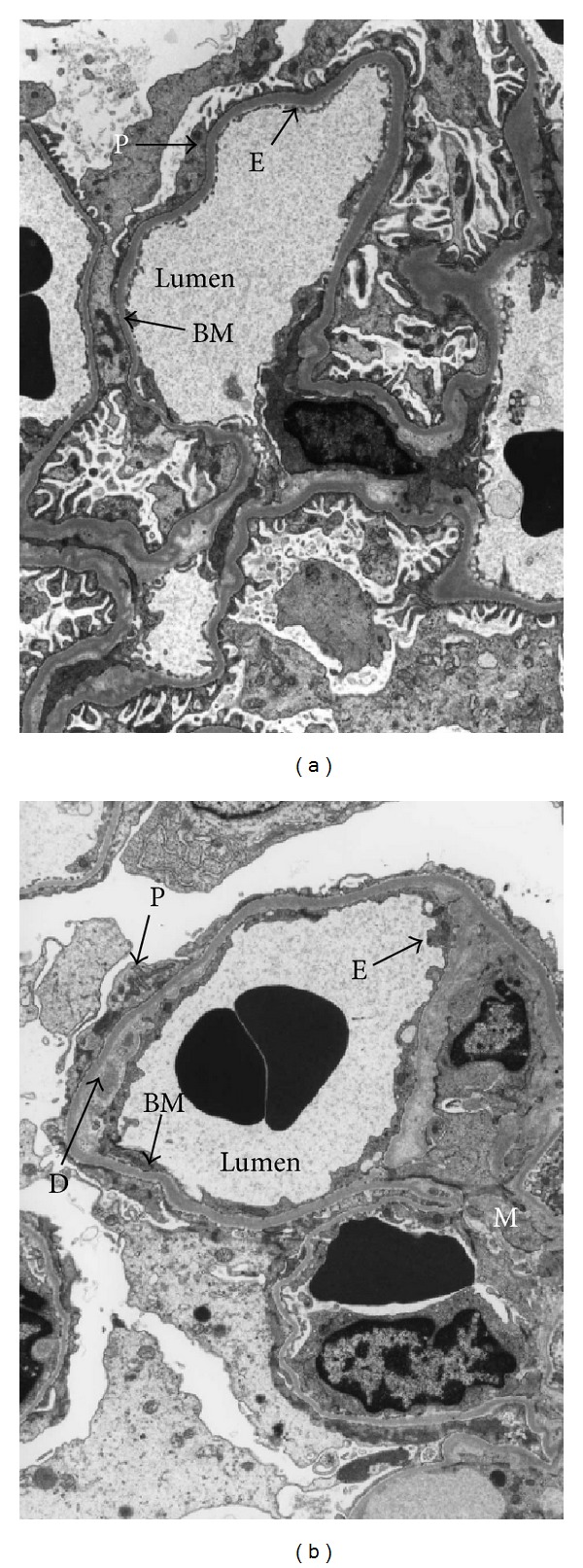
Representative examples of electron micrographs of glomerulus (×7,000) showing (a) isograft with normal glomerular architecture (E: endothelial cell; P: podocytes; Lumen: capillary lumen) and (b) allograft showing new basement membrane (BM), subendothelial deposit (D), mesangial expansion (M), and effacement of foot process (P).

Representative electron micrographs showed a fenestrated endothelial lining in the glomerular capillary, unexpanded mesangium, normal glomerular basement membrane, and intact podocytic foot processes in the isografts (Figure 4(a)). The allograft showed evidence of subendothelial deposits and formation of a new glomerular basement membrane together with the loss of fenestrated endothelium and effacement of podocytic foot processes (Figure 4(b)).

### 3.4. Total TG Enzyme Activity

Total TG enzyme activity, measured in renal homogenates using ^14^C putrescine as the substrate in the presence of calcium, was significantly higher in the allografts than the isografts (1.09 ± 0.13 nmol/h/mg protein versus 0.41 ± 0.03 nmol/h/mg protein, *P* < 0.05).

### 3.5. TG2 Distribution and Quantification

**Figure 5:**
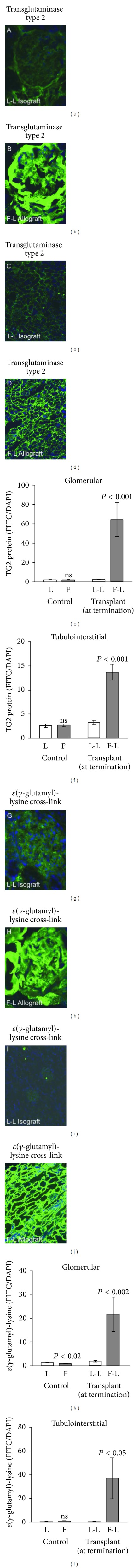
Representative fields of immunofluorescence staining (FITC, green) for transglutaminase type 2 protein ((a)–(d)) and *ε*(*γ*-glutamyl)-lysine ((g)–(j)) for the Lewis- (L-) Lewis (L) isograft and Fisher- (F-) Lewis (L) allograft in both glomerular and tubulointerstitial areas. Transglutaminase type 2 protein ((e)–(f)) and *ε*(*γ*-glutamyl)-lysine cross-link ((k)–(l)) were quantified by multiphase image analysis and expressed as the FITC/DAPI ratio for L-L isografts and F-L allografts at termination and for control F and L rats. Vertical bars indicate ± SEM.

Basal fluorescence for TG2 protein in kidneys obtained from control Fisher and Lewis rats was low, with no significant difference for either glomerular or tubulointerstitial compartments. The allografts showed markedly greater fluorescence for both glomerular (Figures 5(b) and 5(a)) and tubulointerstitial fields (Figures 5(d) and 5(c)), indicating an upregulation of TG2 protein in the allografts. In the isografts at termination, TG2 fluorescence remained similar to values obtained from control Lewis rats. Kidneys from allografts showed a marked increase in the FITC/DAPI ratios for TG2 protein (glomerulus: 64.55 ± 17.61 versus 2.11 ± 0.17, *P* < 0.001, Figure 5(e), and interstitium: 13.72 ± 1.62 versus 3.19 ± 0.44, *P* < 0.001, Figure 5(f)) compared to the isografts, indicating heightened TG2 activity in the extracellular region. A significant, positive linear correlation could be demonstrated between tubulointerstitial TG2 and tubulointerstitial fibrosis (*r* ^2^ = 0.4016, *P* < 0.05).

### 3.6. *ε*(*γ*-Glutamyl)-Lysine Cross-Link Distribution and Quantification

Representative examples are shown for glomerular (Figures 5(h) and 5(g)) and tubulointerstitial fields (Figures 5(j) and 5(i)) for the *ε*(*γ*-glutamyl)-lysine cross-link for isografts and allografts at termination. Basal fluorescence for the *ε*(*γ*-glutamyl)-lysine cross-link obtained from control Fisher and Lewis rats was low in both glomerular and tubulointerstitial compartments. Control Fisher rats showed a significantly lower level of fluorescence in the glomerulus than control Lewis rats although there was no significant difference in the tubulointerstitial compartment. In the isografts, at termination, *ε*(*γ*-glutamyl)-lysine fluorescence was similar to control kidneys. The allografts, however, showed a 10-fold increase in *ε*(*γ*-glutamyl)-lysine fluorescence in the glomerulus (21.75 ± 7.21 versus 1.98 ± 0.37, *P* < 0.005) (Figure 5(k)) and an 80-fold increase in the tubulointerstitium (36.96 ± 17.06 versus 1.88 ± 2, *P* < 0.05) (Figure 5(l)). A significant positive linear correlation could be demonstrated between tubulointerstitial *ε*(*γ*-glutamyl)-lysine and tubulointerstitial fibrosis (*r* ^2^ = 0.5541, *P* < 0.02).

### 3.7. Urinary *ε*(*γ*-Glutamyl)-Lysine Cross-Link

**Figure 6:**
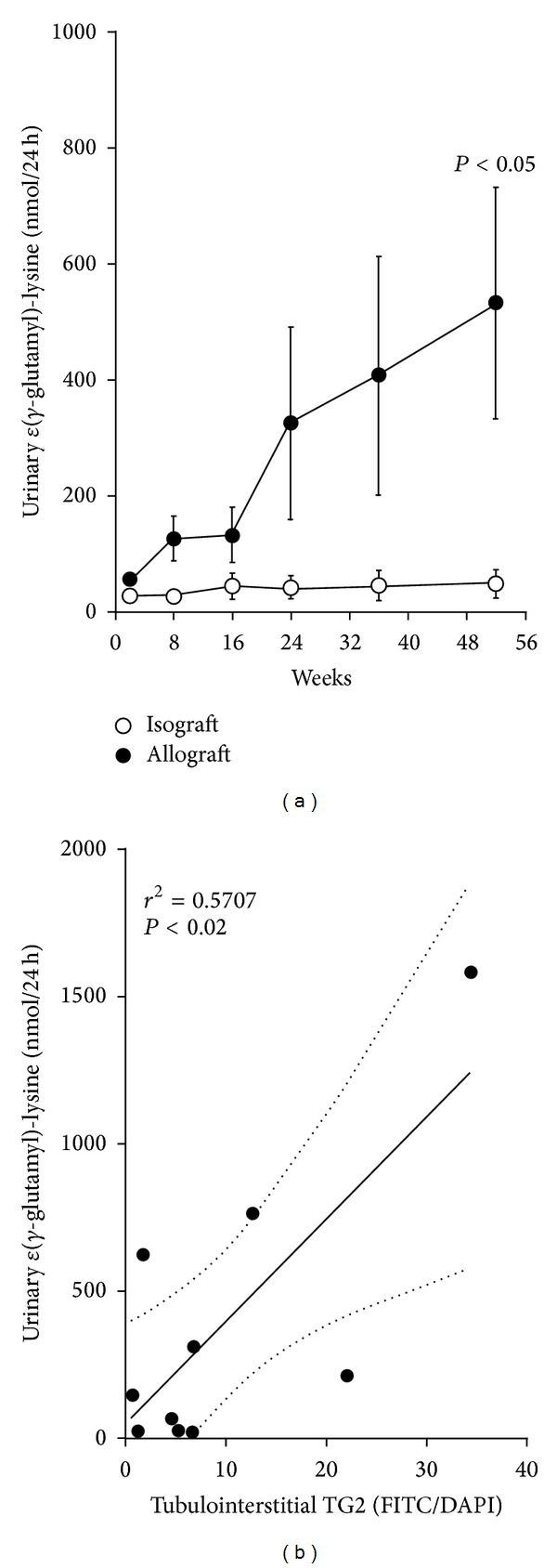
(a) Serial measurements of urinary total *ε*(*γ*-glutamyl)-lysine excretion are shown for isograft and allograft. Vertical bars indicate SEM. (b) Relationship between kidney tubulointerstitial TG2 protein and urinary total *ε*(*γ*-glutamyl)-lysine excretion showing the linear regression (solid line) together with 95% confidence limits (dotted lines).

Total *ε*(*γ*-glutamyl)-lysine excretion in the isograft was 25.9 ± 1.5 nmol/24 h two weeks after transplant and remained essentially unchanged over the study duration (Figure 6(a)). Total *ε*(*γ*-glutamyl)-lysine excretion from the allograft was significantly higher at 57.2 ± 4.1 nmol/24 h (*P* < 0.05), increasing by 9-fold to 534.2 ± 198.4 nmol/24 h at termination. Urinary total *ε*(*γ*-glutamyl)-lysine showed a positive linear correlation with urinary protein excretion (*r* ^2^ = 0.4133, *P* < 0.0001). At termination, total *ε*(*γ*-glutamyl)-lysine excretion showed a positive linear correlation with the level of tubulointerstitial TG2 protein measured by immunofluorescence (*r* ^2^ = 0.5707, *P* < 0.05) (Figure 6(b)).

### 3.8. Northern Blot

**Figure 7:**
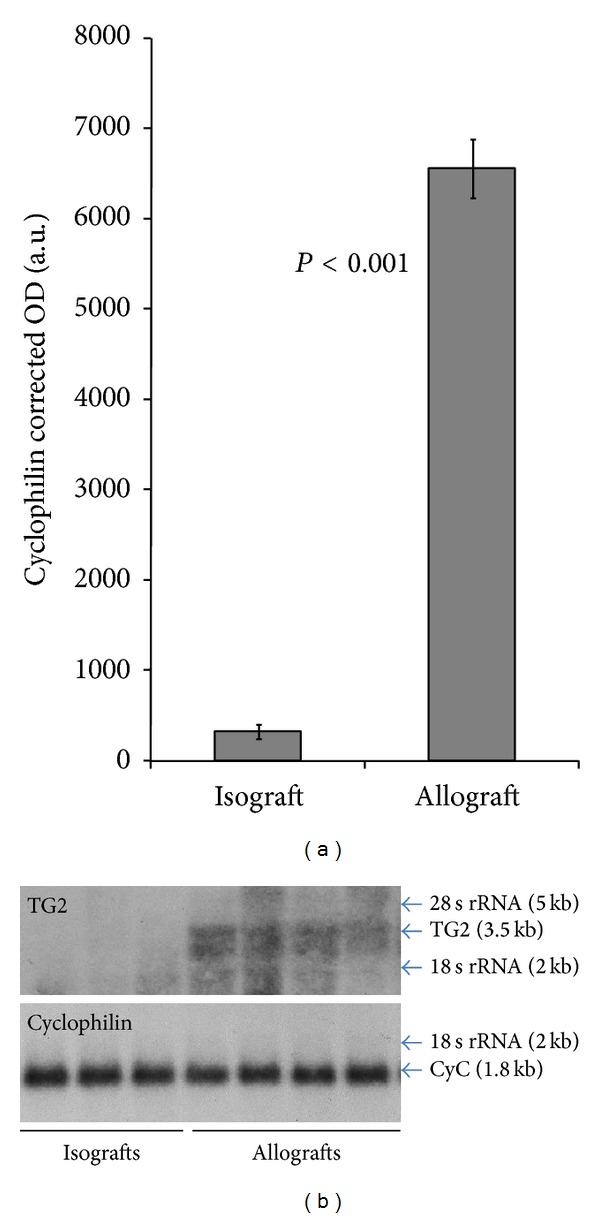
Northern Blot of the renal homogenates for TG2 mRNA. (a) Histogram shows renal TG2 mRNA corrected for loading in arbitrary units of optical density. Vertical bars indicate ±SEM. (b) Autoradiograph showing using cyclophilin as the loading control, isografts (lanes 1–3) and allografts (lanes 4–7).

Northern blot analysis showed a visible band for TG2 mRNA (3.5 kb) derived from renal homogenates obtained from the allografts, which was absent in the renal homogenates derived from the isografts. Using cyclophilin as the loading control (1.8 kb), a 20-fold increase in the volume density of kidney TG2 mRNA was detected in the allografts compared to the isografts (6,523 ± 327 versus 328 ± 77, *P* < 0.001) (Figure 7).

## 4. Discussion

Our study has demonstrated an upregulation of TG2 pathway in the Fisher-Lewis rat RTx model of CAN. Kidneys from control Fisher and Lewis rats contained a similar amount of TG2 protein and *ε*(*γ*-glutamyl)-lysine cross-link in the tubulointerstitium. However, the control Fisher rats had lower levels of the *ε*(*γ*-glutamyl)-lysine cross-link in the glomeruli, which, in the presence of a similar level of TG2 protein, indicated a lower level of glomerular TG2 enzyme activity. No change in the renal TG2 pathway could be detected in the isograft for up to 1 year after RTx. In marked contrast, the allografts showed upregulation of the TG2 enzyme and its cross-link product. Although collagen can autofluoresce, good negative controls with very low background were obtained for the FITC immunofluorescence used to quantify either TG2 protein or the cross-link using cryostat sections. None of the quantified FITC immunofluorescence could be attributed to collagen. Similar results were obtained using Texas red as the fluorochrome.

Total TG2 enzyme activity was increased in kidney homogenates, while mRNA analysis indicated an increase in TG2 transcription. Increased TG2 transcription is the most likely explanation for the increase in TG2 protein which shows greater enzymatic activity to generate more cross-link product. An increase in TG2 was demonstrated in extracellular compartments of both glomeruli and the tubulointerstitium where the enzyme is activated by extracellular calcium [30]. In the glomerulus, TG2 was present, predominantly in the mesangium, associated with grossly scarred glomeruli. With more severe damage, TG2 was also detected in periglomerular areas together with periglomerular fibrosis. The tubulointerstitial staining of TG2 was largely peritubular, with expanded interstitium displaying more intense staining similar to findings observed in human kidney transplant biopsies [24]. El Nahas et al. investigated TG2 and its cross-link product in 23 human kidney allografts during the early posttransplantation period, 8 of which developed chronic allograft nephropathy [24]. A more recent clinical study showed increased TG2 mRNA in protocol biopsies from kidney transplant patients who went on to develop CAN, 6 months after RTx [31].

An association between renal fibrosis and TG2 was first demonstrated in the rat following 5/6 subtotal nephrectomy [21]. Following RTx, ischaemia-reperfusion injury followed by immunological injury could initiate events leading to the upregulation of TG2 transcription within the glomeruli. Vascular injury and atherosclerosis are associated with smooth muscle cell proliferation and the upregulation of TG2 [32, 33]. Proliferating parietal epithelial cells, monocytes, and fibroblasts possibly originating from the transdifferentiation of glomerular epithelial cells [34] may also be a source of TG2.

The distribution of TG2, in peritubular and periglomerular spaces, is similar to the distribution of myofibroblasts, as well as the ECM. Although myofibroblasts contribute to production of ECM, it is the perturbation of TG2 production and its release by tubular cells which leads to the enhanced levels of *ε*(*γ*-glutamyl)-lysine found in renal scarring studies [21, 29]. TG2 is considered to be an ECM-modulating enzyme linked to the actions of transforming growth factor-*β*1 (TGF-*β*1) in the scarred kidney [35, 36]. TG2 may play a key role in matrix storage and in the activation of TGF-*β*1, through the cross-linking of the large latent TGF-*β*1-binding protein to the ECM [37]. The excessive accumulation of TG2 within renal epithelial cells can also lead to cell death through extensive intracellular cross-linking of proteins in the absence of apoptosis. Therefore, the level of TG2 expression and level of activation determine its beneficial effects on healing or detrimental death signals [29, 38].

The allograft also contained elevated levels of the cross-link product, *ε*(*γ*-glutamyl)-lysine, in the tubulointerstitium which showed a significant linear correlation with the development of tubulointerstitial fibrosis. The urinary cross-link expressed per milligram protein doubled from approximately 0.7 to 1.6 nmol/mg; however, we have no data to support the origin of the cross-link identified in the urine. While systemic circulating protein such as albumin remains a possibility this would seem less likely and would need to be intramolecular cross-linking since intermolecular cross-linking would greatly increase molecular weight and reduce oncotic activity. Similar observations have been made in both human chronic kidney disease and experimental rat models of chronic kidney disease [21, 23]. TG2 measurements in terminal kidney tissue were supported by the analysis of total *ε*(*γ*-glutamyl)-lysine cross-link product excreted in the urine. At 2 weeks after RTx, total urinary *ε*(*γ*-glutamyl)-lysine excretion from the allografts was already double that detected from the isograft and showed a progressive increase throughout the study. At termination, total *ε*(*γ*-glutamyl)-lysine excreted in the urine gave a significant positive linear correlation with TG2 protein detected in the kidney. As far as we are aware, this represents the first report of total *ε*(*γ*-glutamyl)-lysine excretion measured in the urine of any animal species.

Interventional studies targeting fibrosis in CAN have previously been directed at modifying the synthesis or the breakdown of matrix proteins [13], inhibition of matrix protein cross-linking, and thereby matrix deposition, may represent a viable alternative. TG inhibitors have been demonstrated to both reduce glucose-stimulated, matrix accumulation in proximal tubular cells* in vitro* [39] and reduce the development of kidney scarring* in vivo* in the hypertensive rat following subtotal nephrectomy [26]. Huang et al. have demonstrated inhibition of TG2 with NTU281 in uninephrectomized streptozotocin-induced diabetic rats, where TG2 inhibition significantly reversed the increased serum creatinine and albuminuria in the diabetic rats and also led to fivefold decrease in glomerulosclerosis and a sixfold reduction in tubulointerstitial scarring [40].

In summary, tubulointerstitial fibrosis is a common final pathway of CAN. In this study, we demonstrated an association between the TG2 enzyme and its cross-link product *ε*(*γ*-glutamyl)-lysine and the development of CAN in the Fisher-Lewis allografts, which has not been studied in animal models of CAN previously. Our report on the urinary excretion of cross-link product *ε*(*γ*-glutamyl)-lysine in this model is the first of its kind. Thus our model provides valuable insight into the inhibition of TG2 by using new pharmacological agents to slow the progression of CAN and the application of urinary *ε*(*γ*-glutamyl)-lysine as a useful tool for monitoring CAN.
